# synr: An R package for handling synesthesia consistency test data

**DOI:** 10.3758/s13428-022-02007-y

**Published:** 2022-11-10

**Authors:** Lowe Wilsson, Tessa M. van Leeuwen, Janina Neufeld

**Affiliations:** 1 Center of Neurodevelopmental Disorders at Karolinska Institutet (KIND), Department of Women’s and Children’s Health, Karolinska Institutet, Solna, Sweden; 2 Department of Communication and Cognition, Tilburg School of Humanities and Digital Sciences, Tilburg University, Tilburg, the Netherlands; 3 Donders Institute for Brain, Cognition and Behaviour, Radboud University, Nijmegen, the Netherlands; 4 Swedish Collegium for Advanced Study, Uppsala, Sweden

**Keywords:** Synesthesia, R, Density-based spatial clustering of applications with noise (DBSCAN), Color analysis

## Abstract

Synesthesia is a phenomenon where sensory stimuli or cognitive concepts elicit additional perceptual experiences. For instance, in a commonly studied type of synesthesia, stimuli such as words written in black font elicit experiences of other colors, e.g., red. In order to objectively verify synesthesia, participants are asked to choose colors for repeatedly presented stimuli and the consistency of their choices is evaluated (consistency test). Previously, there has been no publicly available and easy-to-use tool for analyzing consistency test results. Here, the R package synr is introduced, which provides an efficient interface for exploring consistency test data and applying common procedures for analyzing them. Importantly, synr also implements a novel method enabling identification of participants whose scores cannot be interpreted, e.g., who only give black or red color responses. To this end, density-based spatial clustering of applications with noise (DBSCAN) is applied in conjunction with a measure of spread in 3D space. An application of synr with pre-existing openly accessible data illustrating how synr is used in practice is presented. Also included is a comparison of synr’s data validation procedure and human ratings, which found that synr had high correspondence with human ratings and outperformed human raters in situations where human raters were easily mislead. Challenges for widespread adoption of synr as well as suggestions for using synr within the field of synesthesia and other areas of psychological research are discussed.

## Introduction

### Synesthesia

Synesthesia is commonly described as a phenomenon where a stimulus, referred to as an *inducer*, elicits an experience (*concurrent*), without requiring any conscious effort on the experiencer’s part where most people would not have the same experience under comparable conditions (Grossenbacher & Lovelace, 2001). Many different types of synesthesia exist, but the majority of studies have focused on grapheme-color synesthesia (GCS), where *graphemes* (written symbols, e.g., letters and numbers) trigger color sensations (Ward, 2013). An archetypal example is if a person with GCS always experiences the color red when reading the letter “A” even though it is printed in black.

### Synesthesia-related measures

**Fig. 1:**
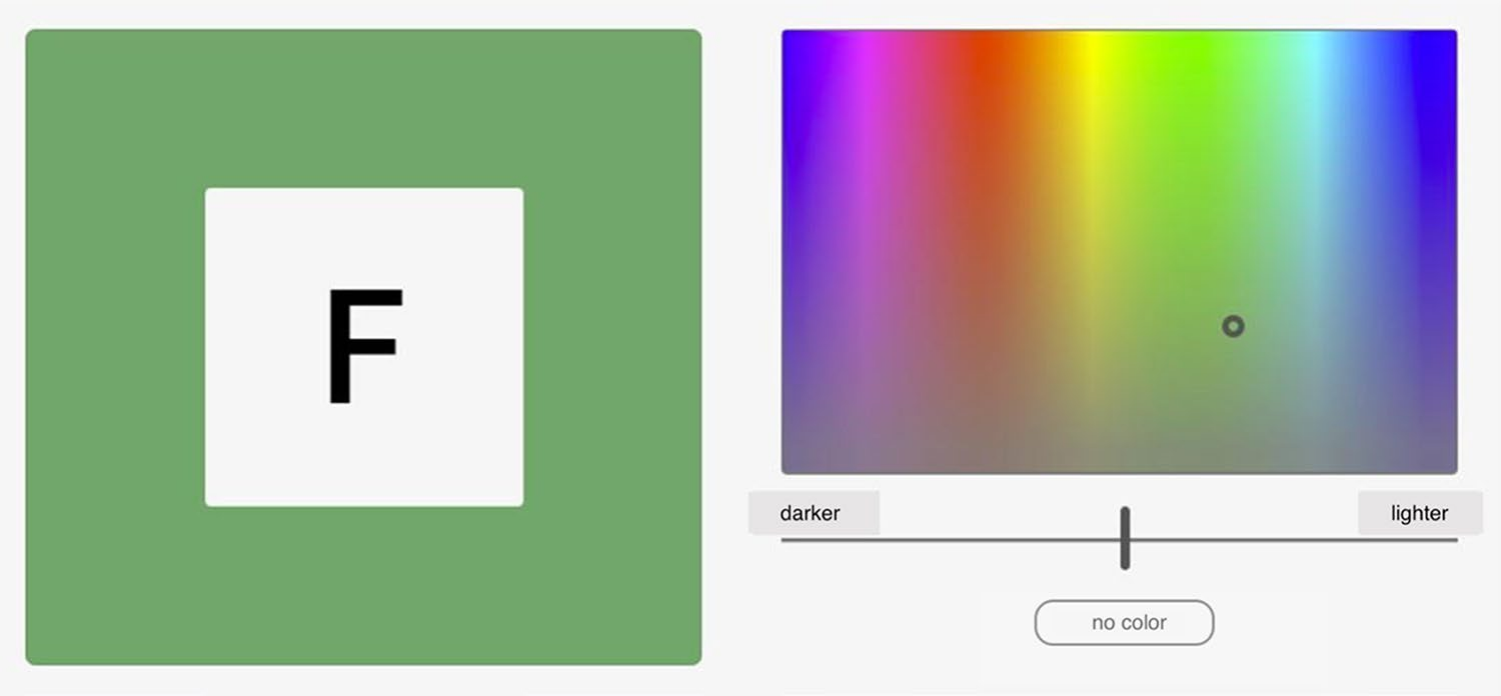
Example of a consistency test trial with inducer “F”. The participant uses the color palette on the right-hand side to choose the color they think best fits “F”

Researchers have investigated synesthesia since at least the nineteenth century, with varying methods for identifying and measuring qualities of the phenomena (Ward, 2013). Over time, subjective self-reports (e.g., Nussbaumer, 1873) have come to be supplemented with objective measures, in order to independently confirm the occurrence of synesthetic phenomena and to investigate them in greater detail. Synesthetic phenomena are highly subjective and varied, but two defining characteristics are the stability (consistency) of synesthetic experiences over time and their automaticity. Thus, Eagleman et al. (2007) developed a standardized test battery that aimed to capture these characteristics. This battery includes a computerized grapheme-color consistency test, based on analog tests which had already been used for decades (Baron-Cohen et al., 1987). Here, graphemes are presented one at a time on a screen and participants are requested to choose, using an in-test color palette, what color they think best “fits” the grapheme (see Fig. 1). Participants are also given the option of selecting “no color”. Each grapheme is randomly repeated three times throughout the test. If the participant is unusually consistent in the colors they choose, this is taken to indicate non-spurious grapheme-color associations. Computerized consistency tests have been used extensively within synesthesia research and have been reported to accurately differentiate self-identified synesthetes from non-synesthetes (Rothen et al., 2013). It should be noted, however, that scoring within the synesthetic range on a consistency test is not sufficient nor necessary for an individual to be identified as synesthete. Subjective experience is a core criterion of synesthesia that needs to be taken into account, and due to the great variability in synesthesia, not all synesthesia types have corresponding standardized consistency tests (Niccolai et al., 2012; Simner, 2012).

### Consistency tests: Variations and challenges

The consistency tests in the battery proposed by Eagleman et al. (2007) include various tests, assessing not only grapheme-color associations, but also word-color (weekdays and months) and sound-color (musical notes and instruments) associations. Consistency test variations applying the same general approach with additional or alternative inducers such as a spoken vowel-color (Cuskley et al., 2019) test have been developed over time.

Apart from varying the format of consistency tests, researchers have also applied different methods for interpreting and scoring test results. Eagleman et al. (2007) suggest a *color variation score*, often referred to as a *consistency score*, based on response colors’ taxicab distances (also called “Manhattan distances”) in RGB color space. Here, lower scores indicate more consistent responses. Eagleman et al. proposed that scores lower than 1 can be considered synesthetic, though they stress that this is “merely an optimal dividing line between two populations whose scores vary along a distribution.” Later studies have sometimes utilized a different color space and/or a different threshold for synesthesia classification. A currently commonly used recommendation is to base consistency scores on Euclidean distances in CIELUV color space and to apply a threshold of 135 (meaning that scores below 135 are taken to indicate synesthesia) in order to optimize the sensitivity and specificity of the consistency score for differentiating synesthetes from non-synesthetes (Rothen et al., 2013). Both Eagleman et al.’s and Rothen et al.’s consistency scoring procedures and thresholds are intended for use with data from consistency tests where each grapheme is presented three times.

Before consistency scores are calculated, it might be advisable to identify and exclude participants with invalid test scores, e.g., who gave too few responses or varied their color choices too little, whether it be on purpose to, say, get through the task quickly or because they misunderstood the instructions. Excluding invalid data might for instance involve removing participants who have used a “no color” button too often (Cuskley et al., 2019) or who responded with the same color for every grapheme (Carmichael et al., 2015; Simner et al., 2006), as identified through visual inspection of responses. Note that there are currently no validated cutoffs regarding the minimum number of chosen colors; researchers decide on a case-by-case basis whether a participant’s data are valid.

One reason why non-synesthetes tend to score above the threshold on consistency tests is that they can usually not memorize all chosen colors for, e.g., 36 graphemes. Choosing the same color for all graphemes does not require much memory effort. Hence, the test cannot differentiate individuals who choose only one or a few colors for strategic reasons or due to misunderstanding the instructions from potential real synesthetes who associate all inducers of a category, e.g., all digits, with the same color. However, past research indicates that synesthetes’ color experiences tend to span a broad color range (e.g., Simner et al., 2005).

In order to identify individuals who use “the same color” too much, time-consuming subjective evaluations are required, which might differ depending on what particular definitions are used for what constitutes different colors. This is made even more difficult if, say, participants should not be allowed to use just two colors for more than 90% of all responses, though such a criterion might be considered reasonable. Taken together, this complicates specifying validation procedures prior to data collection, decreases reproducibility, and might lead to considerable time being spent on data set evaluations in larger studies (or such evaluation being skipped if deemed unfeasible)—since a tool that can detect invalid data automatically is lacking to date.

Though consistency tests are widely used in synesthesia research, a common software tool for the analysis of resulting data has so far not been available. This risks making the field less accessible to newcomers. Moreover, researchers have to either “reinvent the wheel” by implementing procedures for consistency score calculations themselves, or make adjustments to borrowed code to make it work with their own data. This may be time-consuming, and increases the risk of coding errors which might influence analysis results and are perhaps never identified (Soergel, 2014).

### synr: Overview

To tackle the issues described above, an R (R Core Team, 2022) package dubbed synr was developed. R was chosen since it has become increasingly popular amongst researchers and provides sufficient flexibility while being relatively (compared to many general programming languages) easy to learn (Ozgur et al., 2017). synr’s first main purpose is to provide easy-to-learn, efficient and reliable functionality for standard procedures such as consistency scoring. We hope that this will reduce (1) risks of coding errors, (2) barriers of entry to doing research with consistency tests, and (3) the amount of code needed for new projects, saving time and making it easier to share code. Examples of using synr for these standard procedures will follow in the section “Introductory guide to using synr.” synr’s second main purpose is to offer an automated procedure for validating consistency test data, thereby reducing time needed for manually checking data and making validation feasible even for larger data sets.

To enable data validation, i.e., identifying participants with too few or too homogeneous color responses, synr uses two tools for estimating variation in color data. The first tool is a type of cluster analysis (Jain, 2010) algorithm called density-based spatial clustering of applications with noise (DBSCAN; Ester et al., 1996; Khan et al., 2014). Specifically, synr relies on the R package *dbscan* (Hahsler et al., 2019). This enables the identification of clusters (groups) of color responses that are similar to one another (proximal in three-dimensional color space). Colors which do not qualify for any specific cluster, i.e., are not similar (proximal) to a sufficient number of other data points in the data set, are collected in a *noise cluster*. The second tool is a particular measure of spread in three-dimensional (3D) space, dubbed *total within-cluster variance* in synr. This measure produces a value for each participant which relates to the extent to which colors vary within the identified clusters. Both DBSCAN clustering and within-cluster variance calculations are based on a color space (e.g., RGB or CIELUV) selected by the user. Examples of validating data with synr will follow in the section “Introductory guide to using synr”. For more technical details, please refer to Appendix 1 as well as the package’s official help documentation and vignettes (long-form guides), also available at https://CRAN.R-project.org/package=synr.

synr’s functionality is verified with automated tests (Dustin et al., 1999), i.e., computer-run tests which ensure that procedures work as intended. Additionally, synr’s code is openly accessible on GitHub (https://github.com/datalowe/synr). The automated tests reduce the risk of errors to begin with, and anyone is free to double check the code themselves.

Before explaining how synr is used in practice we now turn briefly to a wider discussion about synesthesia, to provide some more context to consistency tests and synr, in particular for researchers outside the synesthesia field.

### Relevance of synesthesia, consistency tests and synr for perception research in general

The brief description of synesthesia presented at the outset of this article leaves many questions unanswered about the phenomenon. For instance, there has been much debate over more specific definitions as well as whether synesthesia is qualitatively different from ordinary perceptual phenomena, or is part of a perceptual continuum (Ward, 2013). This debate also ties into whether individuals can be dichotomously categorized as synesthetes and non-synesthetes, which is what consistency tests have mostly been used for. Some researchers (e. g. Cohen, 2017; Merleau-Ponty, 2011) propose that synesthesia is not a fundamentally distinct perceptual phenomenon that only occurs in particular individuals (synesthetes), and Itoh (2020) argues that any dichotomous separation of perceptual experiences as synesthetic or non-synesthetic is necessarily arbitrary. An important caveat is that even if synesthesia would not be fundamentally different from other perception, there would still be value in characterizing and investigating “typically synesthetic” (Itoh) phenomena as extreme (in the statistical sense) forms of perception, e.g., through the use of consistency tests. In favor of the opposite view, where synesthesia is considered a distinct phenomenon, Ward (2019) proposes three defining characteristics for synesthesia and presents empirical evidence as well as theoretical arguments. Though the matter of the relationship between synesthesia and perception in general has not been settled, it has been shown that findings from synesthesia research can provide insights that are common to perception in everyone (van Leeuwen et al., 2015; Ward et al., 2006). Similarly, tools traditionally used for synesthesia research, including consistency tests and by extension synr, may be employed by researchers for investigating more common stimulus-color associations. Examples of relevant phenomena are vowel-color associations in the general population (Kim et al., 2018) and common experiences of color when viewing objects in dim light, where there is no stimulation of eye cones (Pokorny et al., 2006; Zele & Cao, 2015).

We now proceed with a brief introduction to how synr can be used in practice. Then, we present two example applications of synr. The first one will make use of pre-existing data to demonstrate how synr can be used for standard procedures with a larger data set, and to highlight how synr’s data validation can remove potentially problematic data. The second application will use a smaller set of novel data in order to directly compare synr’s automated data validation with manual validation.

## Introductory guide to using synr

### Installation

For instructions on installing R (R Core Team, 2022) and the widely used R development tool RStudio (RStudio Team, 2022), see their respective references. synr has been accepted by the Comprehensive R Archive Network (CRAN) (R Community, 2022) and can thus be installed in R with the single command install.packages(“synr”). In order to then load its contents, run library(“synr”).

### Loading data

synr expects raw test data to be in a tidy data format (Wickham & Grolemund, 2016) and include participant IDs, which inducer was presented for each trial and participant response colors. Reformatting data to a tidy data format falls outside the scope of synr; Wickham and Grolemund (2016) provide a comprehensive introduction to tidy data and how data can be reformatted with R tools. All color responses that are to be considered invalid and excluded from analysis (e.g., “no color” responses) should be coded as NA (“not available”, “missing”) values. Once a correctly formatted R data frame has been formed, this is passed to synr for the creation of a *participant group* object. synr includes example raw data which can be used thus:

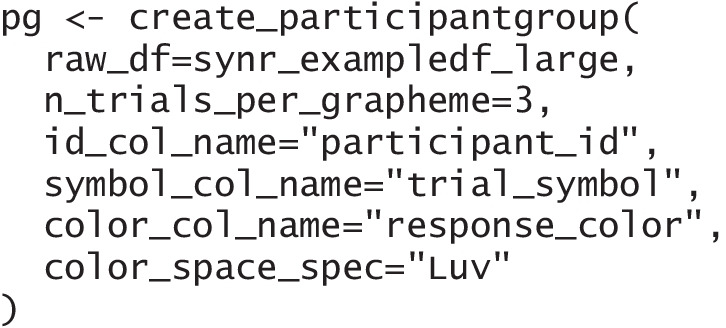



This tells synr (1) that the raw data are stored in an R data frame synr_exampledf_large , (2) that the consistency test had three trials per inducer, (3) where synr should look for the different types of data in the data frame, and (4) the type of color space (here, CIELUV) on which to base coming procedures such as consistency scoring. The resulting participant group object is named pg. It should be mentioned that synr handles the same color spaces supported by base R: standard RGB (sRGB), CIE XYZ, CIELAB and CIELUV. If, instead of “Luv”, color_space_spec=“sRGB” had been specified, all the following operations would be based on sRGB space. For a discussion about the relative merits of the different color spaces, please see Rothen et al. (2013).

Further details about data import are available in the official synr documentation vignette “Creating ParticipantGroup objects”, also available at https://CRAN.R-project.org/package=synr.

### Participant group operations

Most of synr’s procedures are applied by using *methods* linked to the created participant group (pg). Methods are essentially procedures that can reference data embedded in the object. Users do not need to understand this in any detail; interested readers can read more about object-oriented programming (Stefik & Bobrow, 1985) in R (Wickham, 2019). Examples of using participant group methods follow here.

As described in the introduction, consistency scores are commonly calculated for consistency tests. This can be done with the participant group object created earlier as follows:

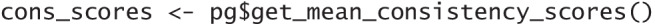



$ is used as a separator between the object pg and the method’s name. This produces a list (vector) of consistency scores, with one score per participant. Instead of looking at all test data, however, one might want consistency scores to be based on a certain subset of data, such as only those from trials involving digit inducers. This requires a slight modification:

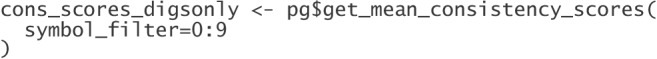



The resulting values may be used with non-synr functions, e.g., calling hist(cons_scores) produces a simple histogram of scores with R’s built-in function hist.

To apply synr’s validation procedure to the example data, producing an R data frame with validity classification data for each participant, the following command may be used:

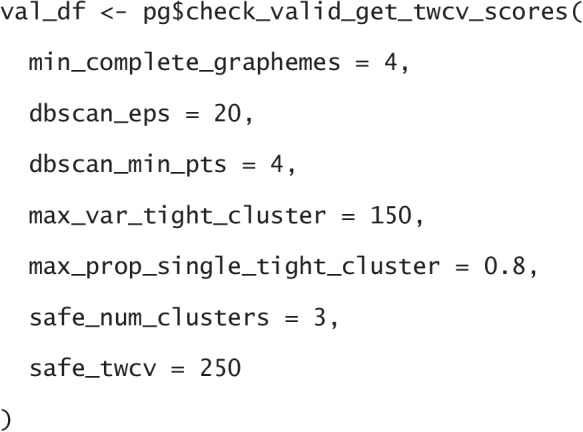



Here, synr is instructed to classify data based on criteria that can be roughly described as “classify data as valid only if there were complete color response data for at least 4 inducers, and at least 3 clearly different colors were used”. These criteria serve only as an example and the corresponding specifications can easily be modified, e.g., the minimum number of required complete color responses can be reduced to 2 by setting “min_complete_graphemes = 2”.

Please note that synr, including its validation procedure, does not treat any region of the selected color space differently from others. For instance, the validation procedure can be used to identify issues with a certain proportion of responses being clustered in any color region, including black. However, since inducers are often shown in black to study participants, some researchers might wish to always exclude all black responses from calculations (e.g., consistency scoring), regardless of how many there are. Since opinions regarding what color space boundaries define “black” will differ, synr does not offer specific functionality for handling this in order to remain unopinionated. Instead, it is recommended that any responses that are to be excluded (similar to “no color” responses) are recoded to NA values before creation of the participant group object. As an example, one could recode any pure black (color hex code “#000000”) response colors to NA in R before handing the data to the create_participantgroup function.

More information about general synr functionality is available in the package vignette “synr: Main tutorial”. This also includes additional methods, such as pg$get_mean_colors() for calculating the mean value for each color axis (e.g., in CIELUV: mean “L”/Lightness, “U” and “V” values). Details about the validation procedure are found in Appendix 1 and the vignette “Validating participant color response data”.

## Example application of synr: vowel-color data

To demonstrate synr’s applicability to larger data sets, especially its automated validation functionality, an example of its use with openly accessible study data (Cuskley et al., 2019) is provided in a synr package vignette (“Using synr with real data: Coloured vowels”; https://CRAN.R-project.org/package=synr), which is briefly described and expanded upon here. The data are from 1164 participants who did a vowel(inducer)-color(concurrent) consistency test, with 16 vowels and three trials per vowel. Vowels were presented as audio recordings, while responses were recorded using an RGB color picker, similar to the one in Fig. 1.

In the vignette, data were used to create a participant group object and calculate consistency scores. The number of vowels that each participant provided three valid color responses for (where “invalid” means missing or “no color” responses) was also calculated, since similar counts were used by Cuskley et al. for data validation. At the end of the vignette, examples of how values produced by synr can be used to replicate some of the graphs in Cuskley et al.’s article are presented. These sections serve to demonstrate that synr can be used, with few lines of code, for common procedures with larger sets of actual consistency test data.

Turning now to data validation, Cuskley et al. describe that they removed participants who gave invalid (“No color”) responses “for more than half of the items in the vowel association task”. Our interpretation of this statement was that a participant’s data need to include at least eight inducers with three valid color responses each to be considered valid, and 59 participants with such invalid data were identified in the vignette.

To identify any clearly problematic additional data sets, synr’s automated data validation was applied in the vignette. The operationalized criteria given to synr for validation here can be roughly described as defining data sets where 80% or more of all response colors were very similar. This resulted in an additional 17 data sets being classified as invalid. Of those, three clearly involve a single color being used (e.g., one participant responded with a red color on every trial). The remaining 14 would not necessarily be deemed as having the same color by a human rater. For a summary of the results, see Table 1.

**Table 1:** Number of valid and invalid data sets in data from (Cuskley et al., 2019) identified with synr, by set of validation criteria. VC1: A participant’s data must include at least eight inducers with three valid color responses each to qualify as valid. VC1+2: Additionally, no more than 79% of the participant’s responses may clearly have the same color (e.g., black). VC1+2+3: Additionally, no more than 79% of the participant’s responses may have colors that are very similar in hue, saturation or brightness (e.g., responding with very light colors, though of different hues)

Validation criteria	Valid data sets	Invalid data sets	Total
VC1	1105 (94.9%)	59 (5.1%)	1164
VC1+2	1102 (94.7%)	62 (5.3%)	1164
VC1+2+3	1088 (93.5%)	76 (6.5%)	1164

**Fig. 2:**
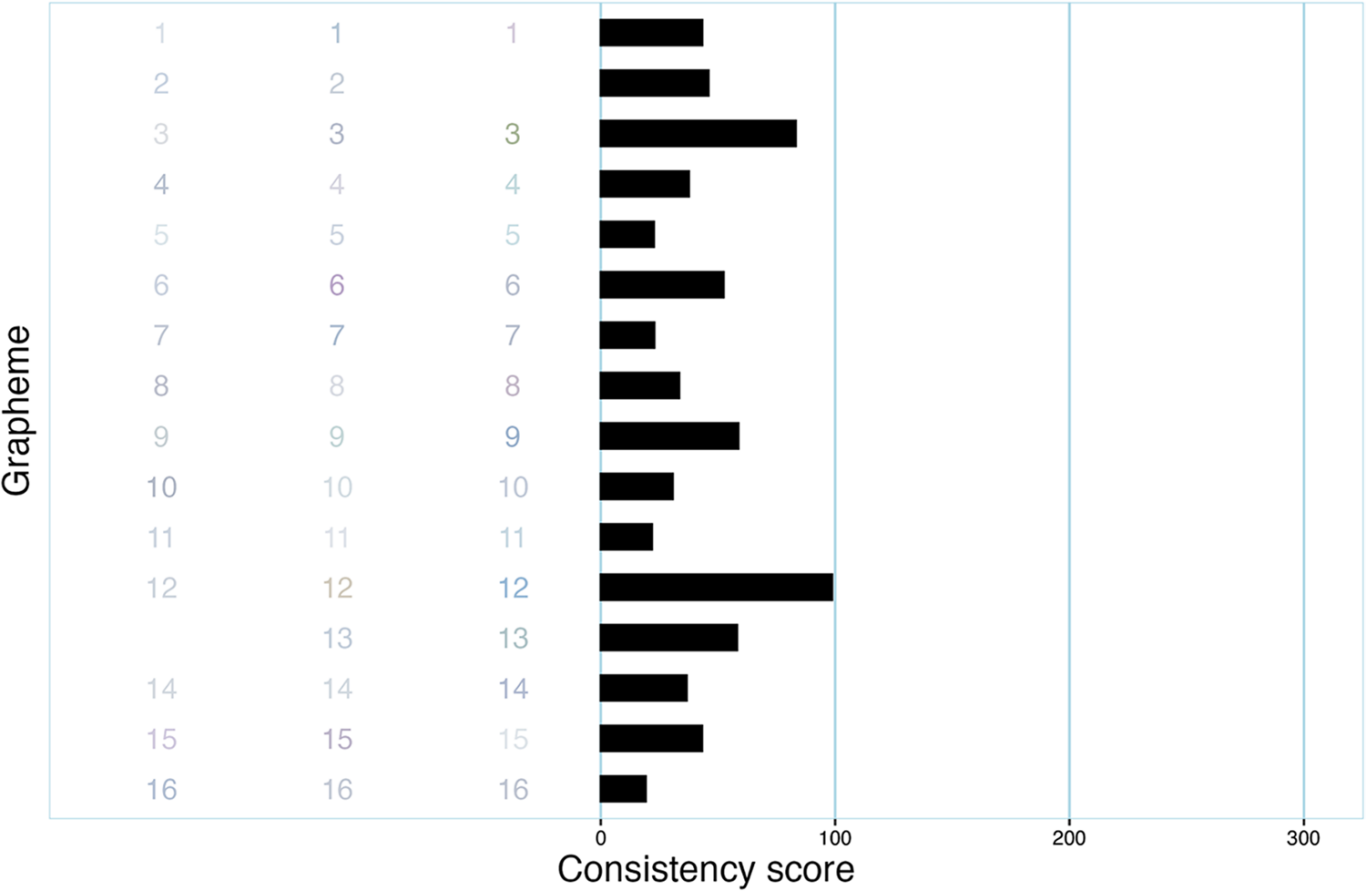
Plot, produced with synr, of problematic vowel-color consistency test data from a single participant. Vowels are represented by numeric codes used in the original article (Cuskley et al., 2019), which are displayed in the color the participant used to respond. For example, on the three trials where the vowel given numeric code “3” was presented, the participant chose colors “very light grey”, “light grey” and “very light olive green”. The bars describe inducer-level consistency scores, which all indicate consistent responses

**Fig. 3:**
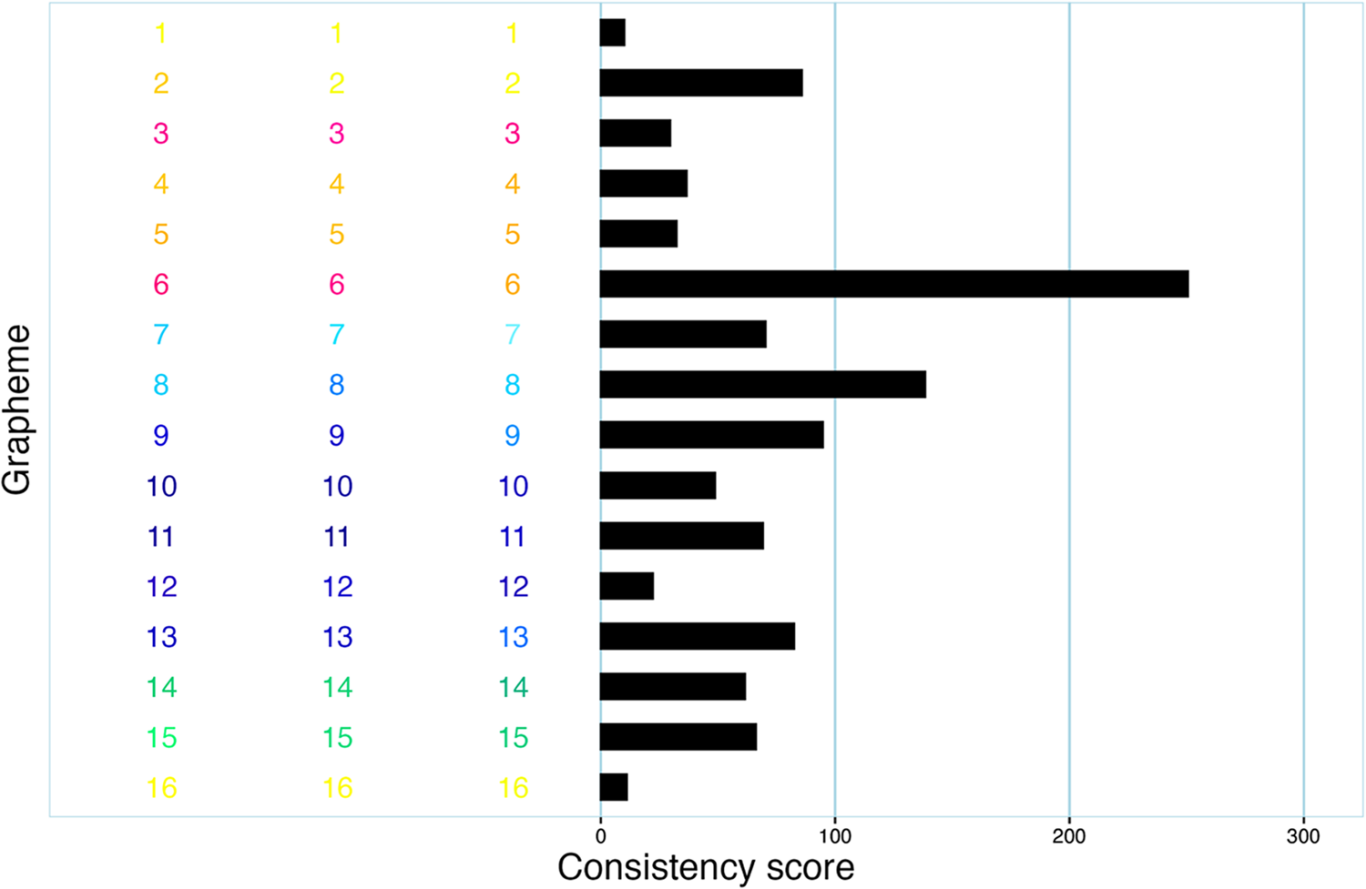
Plot of non-problematic, typical data from a single participant with a consistency score indicating synesthesia, as per Rothen et al. (2013). Refer to Fig. 2 for more information

One of the 17 invalid data sets is illustrated in Fig. 2. As the graph shows, this particular participant noticeably varied the hue of their responses, meaning they used “different colors”. However, all of the colors are very light, as becomes obvious upon comparison with response data that can be considered as typical for a true synesthete (Fig. 3). This means that even though the participant was inconsistent in which hue they picked for a particular vowel, they still got a consistency score of 44.2, thereby qualifying as a synesthete according to the recommended (Rothen et al., 2013) cutoff of 135. Thus, synr is able to identify problematic data beyond the common criteria of participants not giving enough responses or using what a human observer would consider the same color all the time. Naturally, a synesthete may have very light synesthetic colors, but it appears that typical consistency test scoring based on CIELUV color space cannot reliably differentiate such synesthetes from participants randomly selecting very light colors. A similar complication was found by Simner et al. (2017) when basing calculations on RGB color space, as responses involving brighter colors generally lead to participants scoring more consistently, in comparison to responses involving more “dull” colors. In other words, in CIELUV and RGB space; for instance, “light red” is more proximal to “light blue” than “dull blue” is to “dull red”. Thus, a participant’s consistency score can be artificially lowered (indicating more consistency) if they respond with very light colors even if they are inconsistent with regard to color hue. This illustrates that data identified as “invalid” (or “flagged for inspection”, depending on how one uses the results) by synr may sometimes indicate response patterns that are not problematic in themselves, but for which consistency scoring produces no meaningful results. To partly reduce this particular issue, one could base consistency scoring and validation on an alternative color space (in synr, color space is specified when creating a participant group object), though this comes with other problems such as the lack of an established color space-appropriate consistency score cutoff. An example of using alternative color spaces with these data is provided in the supplementary material at https://github.com/datalowe/synr-article-material. It is worth noting, however, that out of all the participants included in Cuskley et al.’s data set, only the example brought up here had a very obvious pattern of “problematically light”, but still varying in hue, responses. This indicates that such cases are unusual.

Cuskley et al.’s article also includes a novel measure of isomorphic cross-modal associations structures based on the Mantel test (Mantel, 1967). In their study, the measure was used to investigate if similar sounds (proximal with regard to their acoustic features, in “acoustic space”) tend to be associated with similar colors (proximal in 3D color space). This goes beyond synr’s current scope, as synr is currently limited to color responses as the dependent variable, and will not be discussed in detail here. However, it is worth noting that the Mantel test-based measure has some parallels to the DBSCAN algorithm used by synr, as the measure also investigates how vowels and colors, respectively, cluster together and form structures. Moreover, similar to synr’s validation procedure, Cuskley et al. note that their measure can identify some “false positive synesthetes”, though the measure was not constructed with this particular aim in mind. Also similar to validation with synr, Cuskley et al. note that this identification is chiefly built on finding participants who are consistent across both trials and items (e.g., all vowels are linked to very similar colors).

## Example application of synr: Data validation with synr compared to human raters

In an ongoing twin study led by one of the authors (Neufeld), online-acquired synesthesia consistency test data sets were all checked for validity manually by two synesthesia researchers (human raters), through visual inspection of graphs presenting participants’ response colors. To evaluate the accuracy of synr’s data validation procedure, synr’s validation procedure was applied with criteria made to match those used by human raters as closely as possible.

### Data characteristics

Participants in the study had previously responded to a short screening measure for synesthesia which contained four items for sequence-color synesthesia (e.g., number-color synesthesia). Only individuals who indicated having had synesthetic experiences on this screening and their twins were eligible for participation in the study, meaning a higher proportion of synesthetes than in the general population was to be expected. The consistency test inducers consisted of written digits, letters (A–Z), weekdays and months (in Swedish). In total, consistency test data were collected from 238 participants.

### Validation procedure

Only grapheme (letters/digits) inducer trial data were evaluated for validity on a per-category basis by human raters. The raters evaluated data independently and used the following validation criteria: (A) The participant must have provided response colors for all three presentations of at least four inducers included in the category in question (e.g., 4 letters). (B) The participant’s responses for inducers in the category must include at least three different colors, where for example different shades of yellow do not count, but orange is regarded as different from yellow or red. (C) The participant must not have used the same color for 60% or more of all their response colors. For criteria B and C, only data related to inducers for which there were three color responses were taken into account. The translation of these criteria into corresponding similar specifications to synr’s validation procedure can be found at https://github.com/datalowe/synr-article-material. Regarding criterion A, note that there is to date no evidence for defining a specific minimum number of inducer-concurrent pairings as a criterion for synesthesia. Hence, defining criteria such as those used here is necessarily an arbitrary choice and will vary depending on the specific research question. For criterion B, it was left to human raters themselves to judge grapheme colors, such as deciding on what was a “shade of yellow” or “orange”, as the main point of this comparison was to see how subjective evaluations by humans would compare to synr’s automated validation procedure.

### Results

Human raters agreed on all but two of the data sets based on consistency test trials with single-letter inducers (99.16% rate of agreement). For digit inducers data, they also disagreed on two data sets. All disagreements resulted from simple mistakes—e.g., two data sets had complete responses for less than four inducers, thereby failing criterion A. Upon reviewing their differences, disagreements were resolved by the raters.

**Fig. 4:**
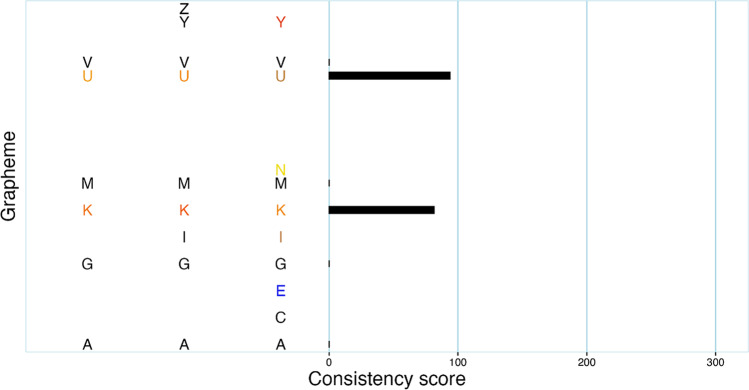
Plot of data set where synr and human raters initially disagreed. Upon review, human raters agreed with synr that more than 60% of the participant’s responses for graphemes with three valid color responses (e.g., K, but not Y) were black. Refer to Fig. 2 for more information on the plot’s formatting. (In order to completely anonymize the data, which letter each set of three response colors is associated with has been randomly rearranged for this graph)

Comparing results from synr’s automated validation with those of human raters (after resolving the raters’ disagreements), there were only disagreements for two-letter-inducers data sets. After reviewing the two data sets, both raters were of the opinion that synr had correctly classified the data sets as invalid, since both participants had used a single color for slightly more than 60% of the trials. One of these two data sets is summarized in Fig. 4.

## Discussion

This article presented synr, an R package implementing standard procedures for analyzing synesthesia consistency test data and a novel approach to automated data validation. Through examples of its application, it was demonstrated that synr may be effectively used with data from consistency tests using different types of stimuli. It was also shown that synr’s validation procedure has a high level of correspondence with manual validation procedures, when validation criteria are correctly operationalized as specifications given to synr. In fact, by avoiding human errors, synr’s validation procedure may be more reliable. Moreover, it provides a way for researchers to operationalize validation criteria without any ambiguity.

Our hope is that synr will be useful for researchers investigating the consistency of stimulus-color associations, whether they consider themselves to be within or outside the field of synesthesia. For example, as mentioned in the introduction, one could repeatedly measure participants’ color experiences in dim light (Pokorny et al., 2006; Zele & Cao, 2015) to see how consistent they are over time for familiar (e.g., a Ferrari car) and novel stimuli (e.g., an object shown in a particular color shortly before test onset). Such novel applications of ideas and tools from the synesthesia research field may be facilitated by user-friendly software by lowering the threshold for other fields’ researchers. In particular, we hope that synr will encourage code sharing, as it offers a concise language for expressing standard procedures such as consistency scoring, with documentation that ensures researchers don’t need to explain R commands in detail. A potential limiting factor for the dissemination of synr is that it requires a certain degree of familiarity with R, which presents a steep learning curve for newcomers to R. On the other hand, learning R in itself brings many opportunities, such as more efficient and reproducible statistical analysis workflows.

synr’s data validation capabilities are likely to be of particular relevance for large-scale studies, where manual validation of all participants’ test data may not be feasible. Technically savvy or interested researchers may study DBSCAN-related literature and synr’s documentation in detail for the sake of fine-tuning the validation. Others may prefer to apply a simpler check, as demonstrated in this article’s first example application, in order to find clearly problematic data sets without delving into the details. To emphasize the importance of checking for invalid data sets, note that Rothen et al. (2013) report a sensitivity of 0.90 and a specificity of .94 when identifying GCS in participants with a consistency test. Estimates of the prevalence of GCS in the general population vary, but as an example, Simner et al. (2006) report a prevalence of 1.1% in a sample of science museum visitors. This prevalence estimate, (0.011) together with Rothen et al.’s sensitivity (0.9) and specificity (0.94) estimates, allows the calculation of a positive predictive value (PPV), the proportion of true positives out of those who test positive (i.e., below cutoff), as follows: $\frac{0.9\times 0.011}{0.9\times 0.011+\left(1-0.94\right)\times \left(1-0.011\right)}\approx 0.143=14.3\%$. The rest of those who test positive would then be false positives. This would mean that, when testing a general population sample with a consistency test fitting the description in Rothen et al. (2013), about 100% − 14.3% = 85.7% of participants identified as having GCS are expected to be false positives. This high rate of false positives may seem counterintuitive due to the relatively high sensitivity and specificity values, but the pattern is very common for identification of rare conditions (Bours, 2021). Either way, the rate indicates a considerable amount of noise that needs to be handled and ideally reduced. Thus, using tools such as automated validation may contribute to improving the reliability of consistency test data analyses, by preventing a subset of false positives and thereby increasing specificity and PPV.

An important limitation regarding consistency test data validation, as discussed in the introductory section, is that there currently is no consensus on validation criteria, such as how many valid color responses a participant must have provided, or how many different colors they must have used at a minimum. Indeed, different validation criteria are likely to be appropriate based on the specific type of synesthesia studied and the research question. Moreover, criteria for specific studies are not always precisely stated. If synr is used on its own for data validation, we highly recommend that the validation code be shared so that readers may see the specifications provided to synr. We also recommend testing validation procedure specifications on a smaller subset of data to see if they seem to be appropriate for the specific study, if possible, in a manner defined upon study preregistration. Users who worry about accidentally labeling good data as invalid with synr may opt for applying a two-step procedure, where after running synr’s validation procedure, an additional manual check is made by human raters. In this case, we recommend that both the validation code and the validation criteria used by human raters be provided with reports of study results, similar to what was done here in the second example application. We hope that synr will contribute to a discussion about standard validation criteria and criteria reporting practices, regardless of whether such criteria would be in terms of synr’s validation functionality or, for example, criteria for human raters. Similarly, future research may investigate how synr’s validation procedure compares to other alternatives, such as approaches building on the structure measure described by Cuskley et al. (2019).

As an open-source project, contributions to synr (at https://github.com/datalowe/synr) are welcome. Researchers are also welcome to translate synr into other programming languages, e.g., Python, or to create their own packages, building on or borrowing ideas from synr. One area that may be particularly interesting is to modify or use parts (conceptual or code) of synr for creating corresponding functionality for data where the dependent variable is not necessarily color-based, but is in an arbitrary 3D space where Euclidean distances are meaningful. An example from the field of synesthesia is tests of sequence-space synesthesia (Rothen et al., 2016; van Petersen et al., 2020; Ward, 2022), where participants are repeatedly asked to place stimuli in a “digital space” based on where they feel the stimuli belong. This type of test also faces the issue of participants who otherwise seem non-synesthetic scoring “consistently” due to low variation in their responses (i.e., selecting responses within a small spatial region; van Petersen et al., 2020; Ward, 2022). With some modification, functionality from synr could be made to handle data from a 3D version of the test and identify such problematic data. Though it would require more comprehensive changes, the same procedures could even be implemented for 2D data, to achieve compatibility with the currently used 2D-based versions of the test (Rothen et al., 2016; van Petersen et al., 2020; Ward, 2022).

## Figures and Tables

**Fig. 5:**
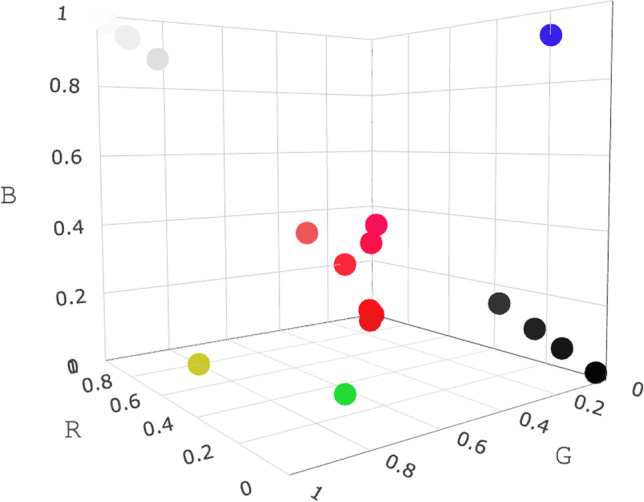
Example of points in RGB color space (values ranging from 0 to 1), where points are colored according to the actual colors they represent. Axis labels: “R”-Red, “G”-Green, “B”-Blue. This plot and Appendix Fig. 6 were produced with the R package plotly (Sievert, 2020). (interactive versions of this plot and Appendix Fig. 6 are available in the synr package vignette “Validating participant color response data”)*.*

**Fig. 6:**
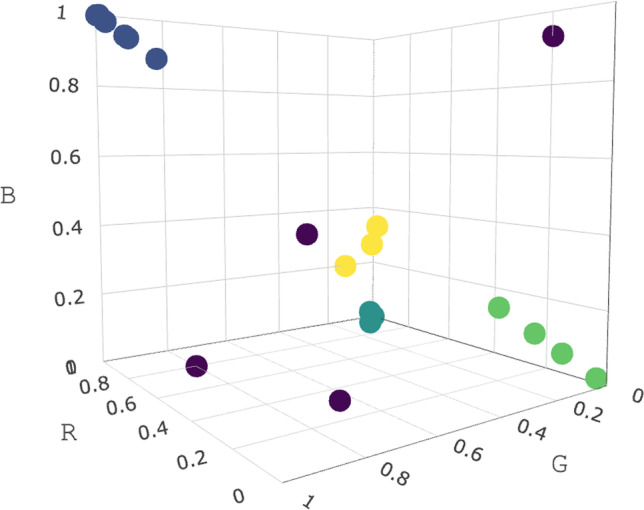
Example of the same points in RGB color space as in Appendix Fig. 5, but where points are colored according to what cluster they were grouped in when given to the DBSCAN clustering algorithm. Identified clusters are now listed. Colored in green, lower right: Near-black points (i.e., close to 0 on all three axes). Colored in dark blue, top left: Near-white points. Colored in turquoise, near center: Intensely red points (high on “R” axis, very low on “G” and “B” axis). Colored in yellow, near center: Less intensely red points (high on “R” axis, low to moderate on “B” axis). Colored in black, spread out: points that were not assigned a cluster (“noise cluster” points).

## Data Availability

The data from the Cuskley et al. (2019) study used for the first example application are available from https://github.com/mdingemanse/colouredvowels. The raw test data that were used for the analysis described in the second example application section unfortunately cannot be shared even in anonymized form due to missing ethics board approval for this. However, some representative mock data and the scripts that were used can be found at https://github.com/datalowe/synr-article-material.

## Appendix 1

### synr’s validation procedure

#### Theory

The validation procedure follows five steps for each participant (i.e., set of color data points), which are described in detail here.

1. **DBSCAN clustering**. Clustering (Jain, 2010) is a general statistical approach for identifying groups of data that belong together and includes a number of specific methods, such as “k-means clustering” and “Density-based spatial clustering of applications with noise” (DBSCAN; Khan et al., 2014). When DBSCAN is applied with a set of points in a space (e.g., a 3D space, such as the RGB color space), it groups together points that are proximal to one another in clusters. Points that are too far from other points to become part of one of the clusters are considered outliers (sometimes referred to as “noise cluster” points). DBSCAN relies on two parameters. The first parameter is the maximum distance allowed between two points for them to be considered neighbors, oft denoted ε, epsilon (or “eps”). The second parameter is the minimum required number of immediate neighbors (at distance ε or closer) of a single point, called a “core” point, for a cluster to start forming (first including the “core” point and all its neighbors), oft denoted “minPts”. Once a cluster has started forming, any point within distance ε from one of the cluster’s points will also be included in the cluster.

To illustrate how DBSCAN clustering can work with color point data, first observe Appendix Fig. 5, a 3D graph of points in RGB color space, colored according to the actual colors they represent. To a typical human observer, it looks like there are groups of black, red and white color responses, respectively (the near-white points in the top-left are hard to make out in the graph because of the white background). Applying DBSCAN with fairly sensible ε (0.15) and “minPts” (3) values, and recoloring color points according to what cluster they are placed in, the result might look like Appendix Fig. 6. Most readers are likely to agree with how DBSCAN clustered together “white” (presented in dark blue in Appendix Fig. 6) and “black” (presented in green) color points, respectively. Depending on how one perceives and defines different colors, one may agree or disagree with how “red” points were grouped into two separate clusters. The point is that using DBSCAN, one can be explicit about how color points should be grouped together, tweaking the ε and “minPts” values as required. Continuing this particular example, if one were to increase the ε value to say 0.25 (from 0.15), all the red points would form a single cluster.

As a sidenote, while synr does give information about how many clusters were identified in participants’ data during validation, it does not include tools for visualizing identified clusters with 3D plots as is done here. Producing such plots is an advanced topic, but interested readers can look at the 3D plot-generating code used in the raw version of the synr vignette “Validating participant color response data”, which is included in the supplementary material at https://github.com/datalowe/synr-article-material.

2. **Calculation of per-cluster centroid.** For each cluster of points, a “center of gravity” or centroid (*x*_*m*_, *y*_*m*_, *z*_*m*_) is produced by calculating the average of all of the cluster’s points’ x, y and z coordinates, respectively. The same is done for the “noise cluster” points.
3. **Calculation of per-cluster “3D variance”.** For each cluster of *n* points, the sum of squared point distances in 3D space between points and their centroid is calculated, then divided by *(n − 1)*: $\frac{\sum_{i=1}^n{\left({x}_i-{x}_m\right)}^2+{\left({y}_i-{y}_m\right)}^2+{\left(z-{z}_m\right)}^2}{n-1}$ . This is arguably analogous to the calculation of variance in one dimension.
4. **Calculation of total within-cluster variance (TWCV).** For each point *p*_*i*_, its squared distance in 3D space from the centroid for the point’s cluster $\left({x}_{m_i},{y}_{m_i},{z}_{m_i}\right)$ is calculated: ${\left({x}_i-{x}_{m_i}\right)}^2+{\left({y}_i-{y}_{m_i}\right)}^2+{\left({z}_i-{z}_{m_i}\right)}^2$. Then, for all *N* points, the point-to-centroid squared distances are summed up and divided by the total number of points minus the number of clusters *C*: $\frac{\sum_{i=1}^N{\left({x}_i-{x}_{m_i}\right)}^2+{\left({y}_i-{y}_{m_i}\right)}^2+{\left({z}_i-{z}_{m_i}\right)}^2}{N-C}$, producing a TWCV value. The reason for subtracting the number of clusters *C* from the total number of points *N* is to account for decreased degrees of freedom. Note that this TWCV concept is, as far as we are aware, new for synr and was decided on following empirical testing similar to what is described in “Example application of synr: Data validation with synr compared to human raters”.
5. **Evaluating user-provided validation criteria.** The results from steps 1–4 are compared with criteria provided by the synr user, as explained in the section discussing parameters below.

A more detailed explanation of how and why this validation procedure was developed is given in the synr package vignette “Validating participant color response data”, also available through https://CRAN.R-project.org/package=synr.

#### synr validation parameters

Recall the validation code example from the “Introductory guide to using synr”:

Note that which color space calculations will be based on is determined by the color space specified when creating the participant group object (CIELUV in the “Introductory guide”). The parameters are now described. “min_complete_graphemes”: The minimum number of graphemes with complete (all non-NA color) responses that a participant’s data must have for them to not directly be categorized as invalid. “dbscan_eps”: Directly corresponds to the DBSCAN clustering algorithm’s ε parameter (see “Step 1” above). “dbscan_min_pts”: Directly corresponds to the DBSCAN clustering algorithm’s “minPts” parameter. “max_var_tight_cluster”: Maximum cluster-level 3D variance (see “Step 3” above) for a cluster to be considered “tight-knit”, i.e., considered to have relatively low variance. “max_prop_single_tight_cluster”: Maximum proportion of points that is allowed to be within a single “tight-knit” cluster (if a participant’s points exceed this limit, their data are classified as invalid). “safe_num_clusters”: Minimum number of identified clusters (“Step 1” above; including the “noise” cluster *if it consists of at least* “dbscan_min_pts” *points*) that guarantees validity of a participant’s data, unless too many points are in a single “tight-knit” cluster. “safe_twcv”: Minimum TWCV (“Step 4” above) score that guarantees validity of a participant’s data, unless too many points are in a single “tight-knit” cluster.

The synr package vignette “Validating participant color response data” provides more details and a more complete usage example.
